# Accuracy in dosimetry of diagnostic agents: impact of the number of source tissues used in whole organ *S* value-based calculations

**DOI:** 10.1186/s13550-020-0614-6

**Published:** 2020-03-19

**Authors:** Anders Josefsson, Klaikangwol Siritantikorn, Sagar Ranka, Jose Willegaignon de Amorim de Carvalho, Carlos Alberto Buchpiguel, Marcelo Tatit Sapienza, Wesley E. Bolch, George Sgouros

**Affiliations:** 1 Russell H. Morgan Department of Radiology and Radiological Science, Johns Hopkins University, School of Medicine, Baltimore, MD USA; 2 Instituto do Cancer do Estado de São Paulo, São Paulo University, School of Medicine, São Paulo, SP Brazil; 3 Department of Biomedical Engineering, University of Florida, Gainesville, FL USA

**Keywords:** Voxelized phantoms, Stylized phantoms, Diagnostic dosimetry, Effective dose, ^68^Ga, DOTA-TATE, ICRP, PET/CT

## Abstract

**Background:**

Dosimetry for diagnostic agents is performed to assess the risk of radiation detriment (e.g., cancer) associated with the imaging agent and the risk is assessed by computing the effective dose coefficient, *e*. Stylized phantoms created by the MIRD Committee and updated by work performed by Cristy-Eckerman (CE) have been the standard in diagnostic dosimetry. Recently, the ICRP developed voxelized phantoms, which are described in ICRP Publication 110. These voxelized phantoms are more realistic and detailed in describing human anatomy compared with the CE stylized phantoms. Ideally, all tissues should be represented and their pharmacokinetics collected for an as accurate a dosimetric calculation as possible. As the number of source tissues included increases, the calculated *e* becomes more accurate. There is, however, a trade-off between the number of source tissues considered, and the time and effort required to measure the time-activity curve for each tissue needed for the calculations. In this study, we used a previously published ^68^Ga-DOTA-TATE data set to examine how the number of source tissues included for both the ICRP voxelized and CE stylized phantoms affected *e*.

**Results:**

Depending upon the number of source tissues included *e* varied between 14.0–23.5 μSv/MBq for the ICRP voxelized and 12.4–27.7 μSv/MBq for the CE stylized phantoms. Furthermore, stability in *e*, defined as a < 10% difference between *e* obtained using all source tissues compared to one using fewer source tissues, was obtained after including 5 (36%) of the 14 source tissues for the ICRP voxelized, and after including 3 (25%) of the 12 source tissues for the CE stylized phantoms. In addition, a 2-fold increase in *e* was obtained when all source tissues where included in the calculation compared to when the TIAC distribution was lumped into a single reminder-of-body source term.

**Conclusions:**

This study shows the importance of including the larger tissues like the muscles and remainder-of-body in the dosimetric calculations. The range of *e* based on the included tissues were less for the ICRP voxelized phantoms using tissue weighting factors from ICRP Publication 103 compared to CE stylized phantoms using tissue weighting factors from ICRP Publication 60.

## Background

Dosimetry for diagnostic agents is performed to assess the risk of radiation detriment (e.g., cancer) associated with the imaging agent. Risk is assessed most commonly by computing the effective dose, *E*. Effective dose is a weighted sum of tissue-equivalent doses, *H*, which themselves are the product of the absorbed dose, *D*(*r*_*T*_), to the target tissue, *r*_*T*_, and a radiation weighting factor, *w*_*R*_; the effective dose coefficient, *e*, is equal to *E* per unit administered activity [1]. In the Medical Internal Radiation Dose (MIRD) committee *S* value methodology, the absorbed dose to a particular tissue is given by the sum, over all source regions, of the time-integrated activity (TIA) assigned to each region (i.e., the source tissue, *r*_*S*_), multiplied by the corresponding source to target S value, *S*(*r*_*T*_ ← *r*_*S*_) [1, 2]. The latter is the absorbed dose to the target per unit TIA in the source tissue. As the number of source tissues used to calculate *e* is increased, the accuracy of the calculation correspondingly increases. There is, however, a trade-off between the number of source tissues and the time and effort required to measure the time-activity curve (TAC) for each tissue needed to compute the TIA. Ideally, all tissues should be contoured in the nuclear medicine image (e.g., SPECT or PET) and their TIA calculated, which is acquired from a sequence of images through time. This is a time-consuming task. Accordingly, the number of source tissues included in a calculation can vary depending on the effort expended on source tissue contouring. Using a previously published comprehensive data set consisting of sixteen patients receiving ^68^Ga-DOTA-TATE, we examine the impact of source tissue number on estimates of the patient’s effective dose coefficient. The analysis was performed using radionuclide *S* values based on Cristy-Eckerman stylized reference phantoms (CE stylized reference phantoms) [3] using tissue-weighting factors, *w*_T_, from the International Commission on Radiological Protection (ICRP) Publication 60 [4], which were compared to the recent ICRP Publication 110 voxelized reference phantom series (ICRP voxelized reference phantoms) and specific absorbed fractions (SAF) from ICRP Publication 133 [5, 6], using *w*_T_ from ICRP Publication 103 [7].

## Methods

The patient data, dosimetric methodologies, and results in this study have been previously described by Josefsson et al. [8]. Abbreviated descriptions are provided below.

### Patients and PET/CT imaging

Sixteen patients, 11 females, and 5 males, from São Paulo, Brazil, underwent two to four whole-body diagnostic ^68^Ga-DOTA-TATE PET/CT scans. The median age for the women in the study was 58 years (age range, 35–79 years) and median weight 75 kg (weight range, 62–109 kg). The men’s median age was 44 years (age range, 36–63 years) and median weight 85 kg (weight range, 68–102 kg). The indications for PET were staging, follow-up, or peptide receptor radionuclide therapy planning of somatostatin avid tumors.

### Dosimetry

Volumes of interest (VOI) were drawn for normal tissues on the PET/CT images 14 organs and tissues: the spleen, liver, kidneys, adrenal glands, brain, heart, lungs, thyroid gland, pituitary gland, salivary glands, testes, red marrow (L1–L5), muscle (right thigh), and whole-body. The salivary glands and pituitary gland are organs that are not represented in the CE stylized reference phantoms. The time-integrated activity coefficients (TIAC) were computed for the whole-body and source tissues by integrating the TAC for each tissue from zero to infinity and dividing with the administered activity. The remainder-of-body TIAC was calculated by subtracting from the whole-body TIAC with the individual TIAC of the respective tissues that were progressively included. To account for the partial volume effects in small organs/tissues (e.g., pituitary gland), a method developed by Plyku et al. [9] for small volumes (e.g., tumors) was used. Regarding larger tissues/organs, the measured activity concentration within the VOIs was used with the respective phantoms tissue/organ to calculate their respective TIAC. To compensate for the size differences between the patient and the respective phantom the TIACs were scaled by the phantom-to-patient whole body mass ratio. Volumes of interest had been drawn of the testes on the male patients but not of the ovaries on the female patients. The average TIAC concentration per unit weight of the testes in the male patients was assumed to be the same for the ovaries in the female patients. The average TIAC of the ovaries (testes and ovaries are represented as the gonads) was calculated as

1 $$ {\mathrm{TIAC}}_{\mathrm{Ovaries}}=\frac{{\mathrm{TIAC}}_{\mathrm{Testes}}}{{\mathrm{Weight}}_{\mathrm{Testes}}}\bullet {\mathrm{Weight}}_{\mathrm{Ovaries}} $$

Dosimetric calculations representing the ICRP methodology were performed using the ICRP Publication 110 voxelized reference phantoms [5], ICRP Publication 133 SAF [6] calculated using ICRP Publication 107 nuclear decay data [10], and ICRP Publication 103 tissue weighting factors [7]. Representing the CE stylized reference phantoms the OLINDA/EXM version 1.0 [11] software was used, which uses tissue weighting factors from ICRP Publication 60 [4] and organ weights from ICRP Publication 89 [12].

The change in effective dose coefficient as the number of source tissues is added was assessed by progressively increasing the number of source tissues added within the dose assessment.

The effective dose coefficient for the CE stylized reference phantoms, *e*_CE_, averaged by the male- and female-averaged patient-specific effective dose coefficients, ${e}_{\mathrm{CE}}^M$ and ${e}_{\mathrm{CE}}^F$ respectively, was calculated according to

2 $$ {e}_{\mathrm{CE}}=\left[\frac{\frac{\sum_{n_M}{e}_{\mathrm{CE}}^M}{n_M}+\frac{\sum_{n_F}{e}_{\mathrm{CE}}^F}{n_F}}{2}\right] $$

where *n*_*M*_ and *n*_*F*_ are the total number of male (*M*) and female (*F*) patients in the study, respectively.

The effective dose coefficient for the ICRP voxelized reference phantoms, *e*_ICRP_, was calculated as

3 $$ {e}_{\mathrm{ICRP}}={\sum}_{r_T}\left[\frac{\frac{\sum_{n_M}{w}_T\bullet {w}_R\bullet d{\left({r}_T\right)}_{\mathrm{ICRP}}^M}{n_M}+\frac{\sum_{n_F}{w}_T\bullet {w}_R\bullet d{\left({r}_T\right)}_{\mathrm{ICRP}}^F}{n_F}}{2}\right] $$

where *d*(*r*_*T*_) is the absorbed dose coefficient (Gy/Bq) for the respective target region, *r*_*T*_. For all ^68^Ga radiation emissions, the radiation-weighting factor, *w*_*R*_ = 1, and the tissue weighting factors, *w*_*T*_, from ICRP Publication 103.

The difference in the percent of the effective dose coefficient, *e*, between including *i* and *i* + 1 number of tissues was calculated as

4 $$ \mathrm{Difference}=\frac{\left({e}_{i+1}-{e}_i\right)}{\left(e-{e}_1\right)}\bullet 100\% $$

Where *e* represent the total effective dose coefficient, and *e*_1_ the effective dose coefficient obtained with the first included tissue/organ.

The stability parameter expressed as the difference in percent between the effective dose coefficient obtained after including all tissues considered, *e*, and the effective dose coefficient, *e*_*i*_, obtained after including *i* number of tissues/organs was calculated as:

5 $$ \mathrm{Stability}=\mathrm{ABS}\left[\frac{\left({e}_i-e\right)}{e}\right]\bullet 100\% $$

The effective dose coefficient calculations were performed for both the CE stylized and ICRP voxelized reference phantoms by incrementing the number of source tissues by (a) source tissue weight (highest to lowest weights), (b) impact (highest increase in percent to lowest), and (c) tissue-weighting factors (highest to lowest).

### Statistical analysis

Statistical analysis was performed using the software Prism version 8.2.1 (GraphPad Software Inc., La Jolla, CA, USA). All data are presented as the mean value ± standard deviation (SD).

## Results

**Fig. 1:**
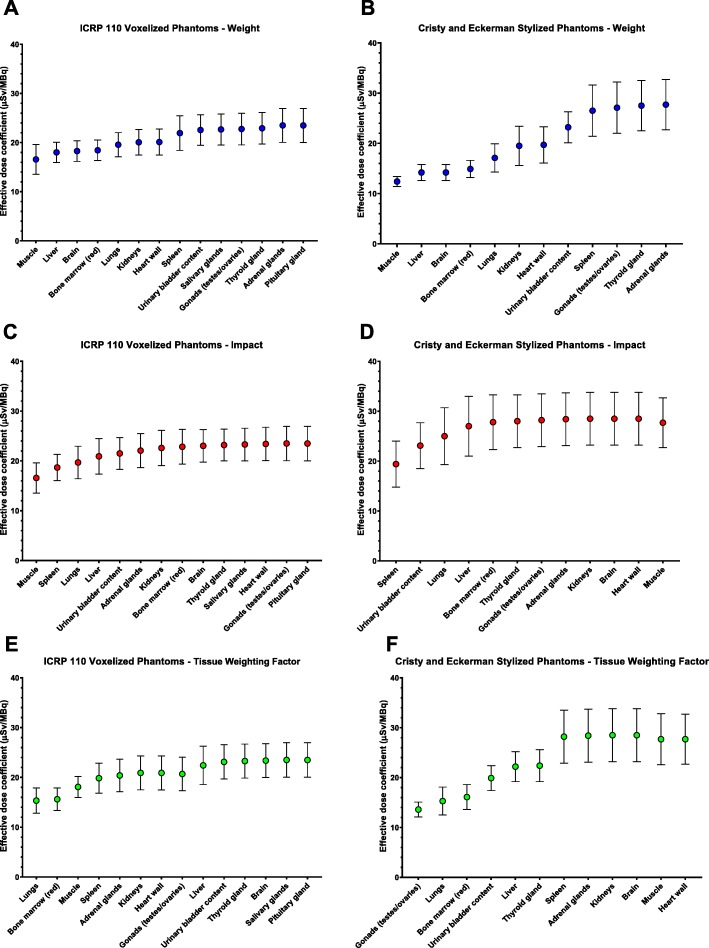
The effective dose coefficient, *e*, in units of μSv/MBq as a function of the number of tissues/organs included in the dosimetric calculations regarding **a**, **b** weight (decreasing); **c**, **d** impact (decreasing); and **e**, **f** tissue weighting factors, *w*_*T*_, (decreasing) for the ICRP Publication 110 voxelized reference phantoms and CE stylized reference phantoms, respectively. The error bars show the standard deviation (SD)

The calculated effective dose coefficient depended upon the order with which source tissues were included in the calculation. One up to 12 source tissues were included for the CE stylized reference phantoms up to 14 for the ICRP voxelized reference phantoms. The effective dose coefficient calculations were performed by incrementing the number of source tissues by (a) source tissue weight (highest to lowest weights), (b) impact (highest increase in percent to lowest), and (c) tissue-weighting factors (highest to lowest). The range of effective dose coefficient for each increment order for the ICRP voxelized and CE stylized reference phantoms, respectively was (a) 16.6–23.5 μSv/MBq and 12.4–27.7 μSv/MBq (Fig. 1a, b, Tables 2 and 5), (b) 16.6–23.5 μSv/MBq and 19.4–27.7 μSv/MBq (Fig. 1c, d, Tables 3 and 6), (c) 15.3–23.5 μSv/MBq and 13.6–27.7 μSv/MBq (Fig. 1e, f, Tables 4 and 7). Using only the whole body TIAC as the remainder-of-body TIAC, the respective effective dose coefficients were 13.9 ± 1.6 μSv/MBq and 14.0 ± 1.7 μSv/MBq for the CE stylized and the ICRP voxelized reference phantoms, respectively. Excluding muscle as a source tissue overestimates the effective dose coefficient with 2.9% (28.5 ± 5.3 versus 27.7 ± 5.0 μSv/MBq) using the CE stylized reference phantoms and underestimates with 3.6% (22.6 ± 4.0 versus 23.5 ± 3.5 μSv/MBq) using the ICRP voxelized reference phantoms. Notably, the effective dose coefficient was a factor 2.0 higher (13.9 ± 1.6 versus 27.7 ± 5.0 μSv/MBq) using the CE stylized reference phantoms when all source tissues and the remainder-of-body TIACs were included, as compared to when the TIAC distribution was lumped into a single reminder-of-body source term. The corresponding result for the ICRP voxelized reference phantoms was a factor 1.6 higher (14.0 ± 1.7 versus 23.5 ± 3.5 μSv/MBq).

## Discussion

The number of source tissues included in phantom-based dosimetry is one of a substantial number of variables that influence the accuracy of the calculation. As the number of source tissues is increased, the pharmacokinetics of the agent within the body is more accurately represented. In 2009, a new reference phantom geometry was made available by the ICRP. The new phantom was derived from a voxelized representation of standard anatomy and includes a close to comprehensive list of source tissues that are represented with greater detail than has been previously available in the CE stylized reference phantom anatomy. Accordingly, the new phantom allows for a more accurate apportionment of the activity distribution. Building upon previous work comparing dosimetry calculations for a PET-imaging agent using the two different phantoms, in this work, we have examined how the effective dose coefficient changes as the activity distribution are made more accurate for both the recent ICRP voxelized reference phantoms and the previous CE stylized reference phantoms.

The CE stylized reference phantoms have up to 28 source tissues and 25 target tissues compared with the ICRP voxelized reference phantoms with up to 76 source tissues and 41 target tissues. Other differences between the ICRP voxelized and the CE stylized reference phantoms are between definitions of different tissues as for example the colon and lungs. In the ICRP voxelized reference phantoms, the colon is divided into three sections the left colon (RC-stem), right colon (LC-stem), and rectosigmoid colon (RS-stem) [5, 6]. In the CE stylized reference phantoms, the colon is divided into two sections the upper large intestine and the lower large intestine [11]. The lungs are represented as one source and target tissue in the CE stylized reference phantoms, in our ICRP voxelized reference phantoms calculations the lungs were represented as one source tissue and four target tissues (bronchi basal cells, bronchi secretory cells, bronchiolar secretory cells, and alveolar-interstitial) [5, 6]. The fractional weights of these four lung tissues were bronchi basal cells (1/6), bronchi secretory cells (1/6), bronchiolar secretory cells (1/3), and alveolar-interstitial (1/3) [6]. The recent ICRP Publication 103, which takes precedence over ICRP Publication 60 has decreased the tissue weighting factors for the gonads (0.20 to 0.08), liver, esophagus, bladder, and thyroid gland (0.05 to 0.04), and increased for the breasts (0.05 to 0.12). In addition, separate values for the brain and salivary glands have been added and the reminder category has increased the number of tissues included as well as the weighting factor itself (0.05 to 0.12) (Table 1) [4, 7]. Previously performed ^68^Ga-DOTA-TATE diagnostic dosimetry published by Walker et al. [13] and Sandström et al. [14], which were calculated using OLINDA/EXM version 1.0 [11] software. The respective reported effective dose coefficients were 26 ± 3 μSv/MBq and 21 ± 3 μSv/MBq, which were based on 6 patients (6 male) and 10 patients (6 male and 4 female), respectively. The effective dose coefficient reference value mentioned by Bodei et al. [15] in current concepts of ^68^Ga-DOTA-TATE PET/CT imaging was that of Sandström et al. [14]. Josefsson et al. [8] calculated the effective dose coefficient 23 ± 3 μSv/MBq using the recent ICRP voxelized reference phantom series [5, 6] and tissue weighting factors from ICRP Publication 103 [7]. This effective dose coefficient was slightly higher, but not significant, and will most likely not have an impact on the ^68^Ga-DOTA-TATE PET/CT imaging protocols used today.

**Table 1:** ICRP Publication 103 [7] and ICRP Publication 60 [4] tissue weighting factors, *w*_*T*_

Tissues/organs	ICRP Publication 103 *w*_*T*_	ICRP Publication 60 *w*_*T*_
Bone marrow (red)	0.12	0.12
Lungs	0.12	0.12
Colon	0.12	0.12
Stomach	0.12	0.12
Breast	0.12	0.05
Gonads	0.08	0.20
Bladder	0.04	0.05
Esophagus	0.04	0.05
Liver	0.04	0.05
Thyroid gland	0.04	0.05
Bone surface	0.01	0.01
Skin	0.01	0.01
Brain	0.01	–
Salivary glands	0.01	–
Remainder tissues	0.12^†^	0.05^*^
∑*w*_*T*_	1.00	1.00

^†^Remainder tissues ICRP Publication 103 (14 in total): adrenals, extrathoracic region, gall bladder, heart, kidneys, lymphatic nodes, muscle, oral mucosa, pancreas, prostate, small intestine, spleen, thymus and uterus/cervix

^*^Remainder tissues ICRP Publication 60 (9 in total): adrenals, brain, small intestine, kidneys, muscle, pancreas, spleen, thymus, and uterus

The results presented will depend upon the agent and its pharmacokinetics within the body. The agent ^68^Ga-DOTA-TATE used in this study showed uptake in the spleen, kidneys, liver, adrenal glands, and pituitary gland and was excreted via the urinary bladder. The number of source tissues that needs to be specified before the effective dose “stabilizes” (comes to within 10% of its final value) may be taken as a measure of whether the pharmacokinetics of the agent in the body is represented adequately. Figure 1 and Tables 2, 3, 4, 5, 6, and 7 show that stability was obtained after 5–9 (36–64%) of the 14 source tissues included for the ICRP voxelized reference phantoms, while stability was reached after 3–9 (25–75%) of the 12 source tissues for the CE stylized reference phantoms. This suggests that the recent ICRP voxelized reference phantoms, which lists more source and target tissues, to reach stability in the optimal order needs more source tissues than the CE stylized reference phantoms. In addition, most dosimetry calculations muscle is not explicitly included as a source tissue. Our results suggest that including this tissue is more important than others for the specific case of ^68^Ga-DOTA-TATE effective dose calculation. This is due to the short physical half-life of ^68^Ga (67.7 min), the circulatory contribution plays a considerable role and the specific uptake contribution is less prominent.

**Table 2:** Impact of number of tissues/organs included based on decreasing weight including blood from ICRP Publication 133, ICRP Publication 110 voxelized reference phantoms and ICRP Publication 103 tissue weighting factors, *w*_*T*_, to calculate the effective dose coefficient, *e*, with the SD. The difference in percent calculated according to Eq. 4 and stability according to Eq. 5

Tissue/organ (*n* = 14)	Weight male/female (g)	Effective dose coefficient *e* (μSv/MBq)	Difference (%)	Stability (%)
Muscle	29,784/17,931	16.6 ± 3.0	–	29.4
Liver	2360/1810	18.0 ± 2.1	20.6	23.3
Brain	1517/1350	18.3 ± 2.1	3.8	22.2
Bone marrow (red)	1394/1064	18.4 ± 2.1	2.4	21.5
Lungs	1200/950	19.6 ± 2.5	16.4	16.7
Kidneys	422/357	20.1 ± 2.6	7.3	14.6
Heart wall	386/291	20.1 ± 2.6	0.6	14.4
Spleen	228/187	21.9 ± 3.5	26.4	6.6
Urinary bladder content	200/200	22.6 ± 3.1	9.0	4.0
Salivary glands	89.0/72.2	22.7 ± 3.2	1.7	3.5
Gonads (testes/ovaries)	37.2/12.6	22.8 ± 3.2	1.2	3.1
Thyroid	23.4/19.5	22.9 ± 3.2	2.2	2.5
Adrenal glands	17.4/15.5	23.5 ± 3.5	8.5	0.0
Pituitary gland	0.6/0.6	23.5 ± 3.5	0.0	0.0

**Table 3:** Impact of number of tissues/organs for ICRP Publication 110 voxelized reference phantoms using ICRP Publication 103 tissue weighting factors, *w*_*T*_, to calculate the effective dose coefficient, *e*, with the SD. The difference in percent calculated according to Eq. 4 and stability according to Eq. 5

Tissue/organ (*n* = 14)	Effective dose coefficient *e* (μSv/MBq)	Difference (%)	Stability (%)
Muscle	16.6 ± 3.0	–	29.4
Spleen	18.7 ± 2.6	30.3	20.5
Lungs	19.7 ± 3.3	14.7	16.2
Liver	20.9 ± 3.6	17.7	11.0
Urinary bladder content	21.5 ± 3.2	8.5	8.5
Adrenal glands	22.1 ± 3.4	8.2	6.1
Kidneys	22.6 ± 3.5	7.6	3.8
Bone marrow (red)	22.8 ± 3.5	3.6	2.8
Brain	23.0 ± 3.3	2.8	2.0
Thyroid	23.2 ± 3.2	2.2	1.3
Salivary glands	23.3 ± 3.3	1.7	0.8
Heart wall	23.4 ± 3.4	1.6	0.4
Gonads (testes/ovaries)	23.5 ± 3.5	1.2	0.0
Pituitary gland	23.5 ± 3.5	0.0	0.0

**Table 4:** Impact of number of tissues/organs for ICRP Publication 110 voxelized reference phantoms depending on decreasing ICRP Publication 103 tissue weighting factors, *w*_*T*_, to calculate the effective dose coefficient, *e* with the SD. The difference in percent calculated according to Eq. 4 and stability according to Eq. 5

Tissue/organ (*n* = 14)	Tissue weighting factor *w*_*T*_	Effective dose coefficient *e* (μSv/MBq)	Difference (%)	Stability (%)
Lungs	0.12	15.3 ± 2.5	–	34.7
Bone marrow (red)	0.12	15.6 ± 2.3	3.2	33.6
Muscle	Remainder (0.12)^†^	18.1 ± 2.1	30.5	23.0
Spleen	Remainder (0.12)^†^	19.8 ± 3.0	21.5	15.5
Adrenals glands	Remainder (0.12)^†^	20.4 ± 3.3	6.7	13.2
Kidneys	Remainder (0.12)^†^	20.9 ± 3.4	6.3	11.0
Heart wall	Remainder (0.12)^†^	20.9 ± 3.4	0.0	11.0
Gonads (testes/ovaries)	0.08	20.7 ± 3.4	-2.6	11.9
Liver	0.04	22.4 ± 3.9	21.2	4.6
Urinary bladder content	0.04	23.1 ± 3.4	8.6	1.6
Thyroid	0.04	23.3 ± 3.4	2.0	0.9
Brain	0.01	23.4 ± 3.4	1.1	0.5
Salivary glands	0.01	23.5 ± 3.5	1.5	0.0
Pituitary gland	–	23.5 ± 3.5	0.0	0.0

^†^The order of the remainder organs/tissues were according to the impact shown in Table 3

**Table 5:** Impact of number of tissues/organs included based on decreasing weight for CE stylized reference phantoms using ICRP Publication 60 tissue weighting factors, *w*_*T*_, to calculate the effective dose coefficient, *e*, with the SD. The difference in percent calculated according to Eq. 4 and stability according to Eq. 5

Tissue/organ (*n* = 12)	Weight male/female (g)	Effective dose coefficient *e* (μSv/MBq)	Difference (%)	Stability (%)
Muscle	28,000/17,000	12.4 ± 1.0	–	55.2
Liver	1910/1400	14.2 ± 1.6	11.6	48.8
Brain	1420/1200	14.2 ± 1.6	0.0	48.8
Bone marrow (red)	1120/1300	14.9 ± 1.7	4.6	46.3
Lungs	1000/800	17.1 ± 2.8	14.3	38.4
Kidneys	299/275	19.5 ± 3.9	15.8	29.7
Heart wall	316/240	19.7 ± 3.6	1.6	28.8
Urinary bladder content	200/200	23.2 ± 3.1	22.9	16.2
Spleen	183/150	26.5 ± 5.1	21.4	4.4
Gonads (testes/ovaries)	39.1/11.0	27.1 ± 5.1	4.3	2.0
Thyroid gland	20.7/17.0	27.5 ± 5.0	2.3	0.7
Adrenal glands	16.3/14.0	27.7 ± 5.0	1.3	0.0

**Table 6:** Impact of number of tissues/organs for CE stylized reference phantoms using ICRP Publication 60 tissue weighting factors, *w*_*T*_, to calculate the effective dose coefficient, *e*, with the SD. The difference in percent calculated according to Eq. 4 and stability according to Eq. 5

Tissue/organ (*n* = 12)	Effective dose coefficient *e* (μSv/MBq)	Difference (%)	Stability (%)
Spleen	19.4 ± 4.6	–	29.9
Urinary bladder content	23.1 ± 4.6	44.8	16.5
Lungs	25.0 ± 5.7	22.3	9.8
Liver	27.0 ± 6.0	24.7	2.5
Bone marrow (red)	27.8 ± 5.5	9.4	0.3
Thyroid gland	28.0 ± 5.3	2.7	1.1
Gonads (testes/ovaries)	28.2 ± 5.3	2.7	2.0
Adrenal glands	28.4 ± 5.3	2.4	2.7
Kidneys	28.5 ± 5.3	0.7	2.9
Brain	28.5 ± 5.3	0.0	2.9
Heart wall	28.5 ± 5.3	0.0	2.9
Muscle	27.7 ± 5.0	− 9.7	0.0

**Table 7:** Impact of number of tissues/organs for CE stylized reference phantoms depending on decreasing ICRP Publication 60 tissue weighting factors *w*_*T*_ to calculate the effective dose coefficient, *e*, with the SD. The difference in percent calculated according to Eq. 4 and stability according to Eq. 5

Tissue/organ (*n* = 12)	Tissue weighting factor *w*_*T*_	Effective dose coefficient *e* (μSv/MBq)	Difference (%)	Stability (%)
Gonads (testes/ovaries)	0.20	13.6 ± 1.5	–	51.0
Lungs	0.12	15.3 ± 2.8	12.5	44.6
Bone marrow (red)	0.12	16.1 ± 2.5	5.4	41.8
Urinary bladder content	0.05	19.9 ± 2.5	27.0	28.1
Liver	0.05	22.2 ± 3.0	15.9	20.0
Thyroid gland	0.05	22.4 ± 3.2	1.4	19.2
Spleen	Remainder (0.05)^†^	28.2 ± 5.3	41.6	2.0
Adrenal glands	Remainder (0.05)^†^	28.4 ± 5.3	1.4	2.7
Kidneys	Remainder (0.05)^†^	28.5 ± 5.3	0.4	2.9
Brain	Remainder (0.05)^†^	28.5 ± 5.3	0.0	2.9
Muscle	Remainder (0.05)^†^	27.7 ± 5.1	− 5.4	0.1
Heart wall	–	27.7 ± 5.0	− 0.2	0.0

^†^The order of the remainder organs/tissues were according to impact (percentage increase) shown in Table 6

A total of 14 source tissues were included in the dosimetric calculations using the ICRP voxelized reference phantoms compared with 12 for the CE stylized reference phantoms. The pituitary and the salivary glands were not included as either source or target tissues in the CE stylized reference phantoms as they are in the ICRP voxelized reference phantoms. A common way to perform dosimetric calculations for tumors or small organs (e.g., pituitary gland or salivary glands) has been to use the sphere model, which is included in the OLINDA/EXM version 1.0 [11] software. The pituitary gland has high uptake and the second-highest calculated absorbed dose coefficient previously reported by Josefsson et al. [8]. The pituitary gland did not notably affect the calculated effective dose coefficient for the ICRP voxelized reference phantoms (Tables 2, 3, and 4, Fig. 1a, c, e), which is mainly due to it not being considered a tissue at risk by the ICRP and thus it has not been assigned a unique tissue weighting factor. The pituitary gland has a relatively small TIAC compared to the brain (factor of 10 difference), which is the closest neighboring tissue with a dedicated tissue weighting factor (Table 1), resulting in a low reciprocal contribution. Regarding the CE stylized reference phantoms represented by OLINDA/EXM version 1.0 [11] software, the pituitary gland absorbed the dose coefficient was calculated using the built-in sphere model, which only considers the self-contribution and no contributions from neighboring tissues. In the ICRP voxelized reference phantoms, the pituitary gland is a designated source and target tissue so contributions from neighboring tissues are properly taken into account. In addition, the salivary glands have a tissue weighting factor in the recent ICRP Publication 103 (Table 1) and contributes to the total effective dose coefficient (Tables 2, 3, and 4, Fig. 1a, c, e).

There was no specific uptake noted in the ovaries on the PET-images, nor have any references been found in the literature to indicate that there would be specific uptake with ^68^Ga-DOTA-TATE. An assumption was made based on the uptake in the testes, which was based on the average TIAC per unit weight of the testes and calculated according to Eq.1. This assumption dedicating a specific TIAC to the ovaries was only made to have a source-target for gonads (testes and ovaries) in the calculations, which has a dedicated tissue weighting factor in both ICRP Publications 60 and 103. This increased the effective dose coefficient by 1.7% and 0.1% for the CE stylized and ICRP voxelized reference phantoms, respectively relative to the values published by Josefsson et al. [8].

The ICRP voxelized reference phantoms were made to represent the average adult female and male, and have the respective whole body weights 60 and 73 kg. The variation of the patient’s whole body weights as previously mentioned varies between 62–109 kg for females, and 68–102 kg for males in this study. Regarding normal organs (e.g., liver) the median liver weight was 1.67 kg (weight range 1.04–2.25 kg) in the female and 1.97 kg (weight range 1.42–2.28 kg) in the male patients compared to 1.81 and 2.36 kg, for the adult female and male ICRP reference phantoms liver weights, respectively. This indicates that both the ICRP reference phantoms total body and liver weights are different compared to the patients in this study. These differences between the individual patient and respective reference phantom size will not give individual correct *S* values regarding self- or reciprocal contributions to neighboring organs/tissues. To compensate for the difference in size between the patient and their respective reference phantom, Josefsson et al. [8] normalized the TIACs using the reference phantom and patient total body weight ratio. The next step is to evaluate the non-uniform rational B-spline (NURBS) and polygon mesh (PM) surface-based computational hybrid phantoms developed by the University of Florida [16, 17].

In conclusion, to perform the most accurate dosimetric calculation, all the tissues should be considered. In effective dose calculations, stability (i.e., a difference less than 10% in the effective dose coefficient) was achieved when a minimum of 5 tissues and the reminder-of-body were included for the ICRP voxelized reference phantoms (Table 3, Fig. 1c), and 3 tissues for the CE stylized reference phantoms (Table 6, Fig. 1d). The large tissues (e.g., muscle and liver) and those with high uptake (e.g., spleen), which will be agent dependent, were the most important to include in this particular radiopharmaceutical example.

## Conclusions

This study shows the importance of specifically including large tissues such as the muscles as a source term, along with the remainder-of-body to calculate the effective dose coefficient. The effective dose coefficient range based on the tissues included was less for the recent ICRP 110 voxelized reference phantoms than for the CE stylized reference phantoms. The term stability was introduced (i.e., come to within 10% of the effective dose coefficient when all source tissues are explicitly considered). The number of organs/tissues required to reach stability depended upon the order they were included. The optimal order to reach stability required less organs/tissues using the CE stylist reference phantoms than the ICRP 110 voxelized reference phantoms. This suggests that the recent more realistic ICRP 110 voxelized reference phantoms combined with the latest ICRP 103 tissue weighting factors require more organs/tissues included in the dosimetric calculations to reach stability.

## Data Availability

The datasets used and/or analyzed during the current study are available from the corresponding author on reasonable request.

## Abbreviations

*r*_*S*_: Source region
*r*_*T*_: Target region
*w*_*R*_: Radiation weighting factor
*w*_*T*_: Tissue weighting factor
CE: Cristy and Eckerman
CT: Computed tomography
DOTA: 1,4,7,10-Tetraazacyclododecane- N,N′,N′′,N′′′-tetraacetic acid
ICRP: International Commission on Radiological Protection
MIRD: Medical internal radiation dose
PET: Positron emission tomography
PET/CT: Positron emission tomography/computed tomography
SD: Standard deviation
TAC: Time-activity curve
TATE: (Tyr^3^)-octreotate
TIA: Time integrated activity
TIAC: Time integrated activity coefficient
VOI: Volume of interest
*D*(*r*_*T*_): Absorbed dose to target region
*E*: Effective dose
*H*: Equivalent dose
*S*(*r*_*T*_ ← *r*_*S*_): *S* value source region to target region
*d*(*r*_*T*_): Absorbed dose coefficient to target region
*e*: Effective dose coefficient
